# 2311: Coronary artery calcification on nongated CT scan predicts mortality and acute myocardial infarction after sepsis

**DOI:** 10.1017/cts.2017.128

**Published:** 2018-05-10

**Authors:** Vedant Arun Gupta, Matthew Sousa, Rahul Annabathula, Steve Leung, Vincent L. Sorrell

**Affiliations:** Center for Clinical and Translational Science, University of Kentucky, Lexington, KY, USA

## Abstract

OBJECTIVES/SPECIFIC AIMS: Cardiac complications are common after hospital admission for sepsis, and elevated troponin has been associated with increased all-cause mortality. However, little is known about clinical or imaging factors that predict these cardiac events. Coronary artery calcification (CAC) is an easily identifiable imaging finding, even on nongated CT scans. The goal of this study is to identify if CAC predicts all cause mortality and acute myocardial infarction. METHODS/STUDY POPULATION: This is a single center, nonconcurrent cohort study including 899 patients who were admitted for sepsis and had a detectable TnI level from January 2013 to December 2013. Patients with a CT scan of the chest or abdomen done for other clinical indications within 6 months of this admission were reviewed for the presence or absence of CAC. Medical records were individually reviewed for mortality and type I acute myocardial infarctions at 1 year. RESULTS/ANTICIPATED RESULTS: In total, 144 patients (mean age 57±14.8 years, 48% female) were included in the analysis. CAC was seen in 59% of these scans. Compared to those without detectable CAC, the CAC group had similar APACHE score (18 vs. 16.6, *p*=0.259), peak TnI (3.64 vs. 2.11 mg/dL, *p*=0.363), aspirin (63% vs. 51%, *p*=0.144), and β blocker use (90% vs. 85%, *p*=0.357) and had higher statin use (48% vs. 27%, *p*=0.013). CAC was associated with increased all-cause mortality (59.5% vs. 38.9%, *p*=0.016) and type I myocardial infarctions (10.6% vs. 1.7%, *p*=0.039) compared with those without CAC. DISCUSSION/SIGNIFICANCE OF IMPACT: Coronary artery calcification is often seen when patients present with a noncardiac acute illness, such as sepsis, often making a new diagnosis for these patients. Mortality and acute MI after sepsis can be predicted by coronary calcification, and identify patients who should be targeted for therapy and close follow-up.

